# Circular RNA circRUNX1 promotes papillary thyroid cancer progression and metastasis by sponging MiR-296-3p and regulating DDHD2 expression

**DOI:** 10.1038/s41419-020-03350-8

**Published:** 2021-01-21

**Authors:** Junjie Chu, Li Tao, Teng Yao, Zizheng Chen, Xiaoxiao Lu, Li Gao, Liang Fang, Jian Chen, Gaofei He, Shuying Shen, Deguang Zhang

**Affiliations:** 1 Department of Head and Neck Surgery, Institute of Micro-Invasive Surgery of Zhejiang University, Sir Run Run Shaw Hospital, Medical School, Zhejiang University, Hangzhou, People’s Republic of China; 2 Department of Orthopaedic Surgery, Sir Run Run Shaw Hospital, Zhejiang University School of Medicine & Key Laboratory of Musculoskeletal System Degeneration and Regeneration Translational Research of Zhejiang Province, 3 east Qingchun road, Hangzhou, Zhejiang Province 310016 People’s Republic of China

**Keywords:** Thyroid cancer, Oncogenes

## Abstract

Papillary thyroid cancer (PTC) has a continuously increasing incidence and imposes a heavy medical burden to individuals and society due to its high proportion of lymph node metastasis and recurrence in recent years. Circular RNAs, a class of noncoding RNAs, participate in the progression of many cancers, but the role of circRNAs in PTC is still rarely reported. In this study, circRNA deep sequencing was performed to identify differentially expressed circRNAs in PTC. CircRUNX1 was selected for its high expression in PTC, and circRUNX1 silencing was directly associated with the week potential for migration, invasion and proliferation of PTC in vivo and in vitro. Fluorescence in situ hybridization (FISH) was further used to confirm the cytoplasmic localization of circRUNX1, indicating the possible function of circRUNX1 as a ceRNAs in PTC progression through miRNA binding. MiR-296-3p was then confirmed to be regulated by circRUNX1 and to target DDHD domain containing 2 (DDHD2) by luciferase reporter assays. The strong antitumor effect of miR-296-3p and the tumor-promoting effect of DDHD2 were further investigated in PTC, indicating that circRUNX1 modulates PTC progression through the miR-296-3p/DDHD2 pathway. Overall, circRUNX1 plays an oncogenic role in PTC and provides a potentially effective therapeutic strategy for PTC progression.

## Introduction

Thyroid cancer is currently the most common malignant tumor in the endocrine system. In the past few decades, the incidence of thyroid cancer has increased worldwide year by year, owing to the advancement of medical imaging and the popularity of screening^1^. Papillary thyroid cancer (PTC) is the main subtype of thyroid cancer, with an approximately 85% proportion of cases^2–4^. PTC is now generally accepted to have an ideal prognosis outcome after standardized treatment. However, its high proportion of lymph node metastasis and recurrence^5^ and the low 5-year survival rate of advanced stage patients^6^ are indicators of the severity of the disease. Hence, further exploration of the underlying molecular mechanisms of PTC is particularly significant for the establishment of novel treatments.

MicroRNA (miRNA) is a subgroup of endogenous non-proteincoding single-stranded RNAs with 19–24 nucleotides in length and usually negatively regulate their target gene expression by binding primarily to the 3′-UTR of messenger RNA (mRNAs)^7^. Several miRNAs can act as oncogenic or suppressive factors involved in the progression of PTC^8–10^ and are getting more and more attention recent years.

Circular RNA (circRNA) is an endogenous noncoding RNA discovered in recent years that is mainly produced by back-splicing and differs from the classic 5′−3′ pattern of linear RNA. It exhibits many interesting characteristics: high abundance in eukaryotic cells, evolutionary conservation, high structural stability, etc^11–13^. According to previous studies, circRNAs have various functions related to the occurrence and development of cancer. There is now overwhelming evidence that circRNA which is enriched in miRNA-binding sites can exerts its biological function as competing endogenous RNAs (ceRNAs) and competitively reduce the content of active miRNA^14,15^. Besides, circRNA can interact with different RNA binding proteins to act as protein sponges^16^ or enhance protein function^17^ and can even undergo independent translation under certain conditions^18^. Through these mechanisms, circRNAs regulate the cell cycle, apoptosis, and gene expression^19^. A growing number of studies have shown that variations in the levels of circRNAs are closely related to the development of many types of cancer, such as gastric cancer, osteosarcoma, and hepatocellular carcinoma^20–22^. Nevertheless, we still do not know much about the relationship between the progression of PTC and circRNAs.

In this study, through high-throughput sequencing we identified a novel circRNA, circRUNX1, originating from exons of the runx family transcription factor 1 (RUNX1), with the circBase ID hsa_circ_0002360. Our findings revealed that the level of circRUNX1 was significantly elevated in PTC tissue and correlated with advanced clinical stage, extrathyroidal extension levels and lymph node metastasis. The proliferation, migration, and invasiveness of PTC cells were found to be regulated by circRUNX1 in our studies. Taken together, the results revealed a novel biomarker panel consisting of the circRUNX1/miR-296-3p/DDHD2 axis that is critical in PTC tumorigenesis and invasiveness and may be a novel therapeutic target to intervene in PTC progression.

## Results

### CircRNA expression patterns in human PTC and paracarcinoma tissues

**Fig. 1:**
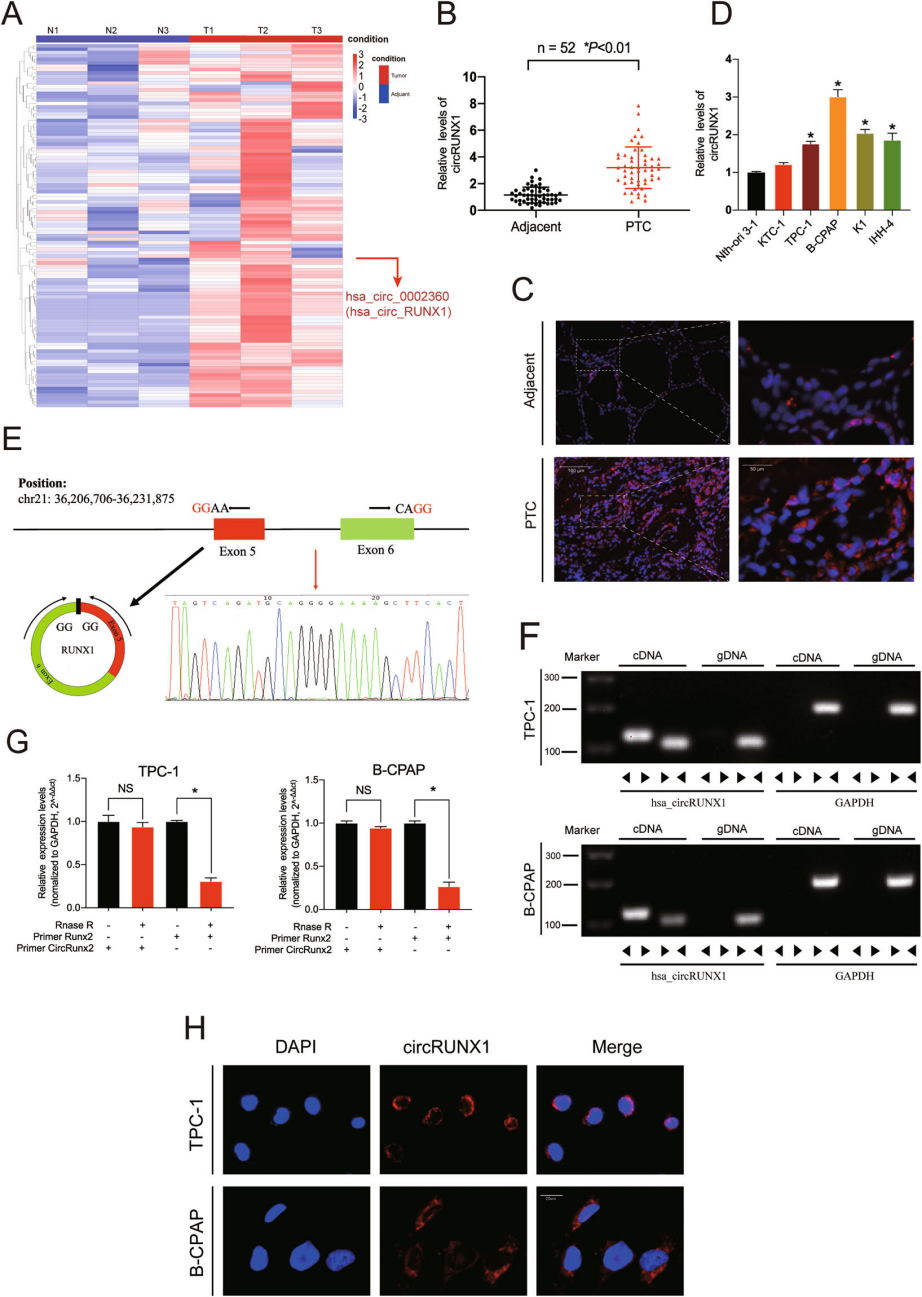
Validation and expression of circRUNX1 in papillary thyroid cancer tissues and cells. **a** Heatmap of upregulated differentially expressed circRNAs based on three pairs of representative PTC and paracarcinoma tissues (Top 100). **b** High levels of circRUNX1 are expressed in human PTC tissues compared with adjacent normal tissues. Data represent the mean ± SD (*n* = 52). **c** FISH assay indicated that circRUNX1 expression was higher in PTC than in paracarcinoma tissues. Representative images are shown. Scale bars = 100 μm or 50 μm. **d** CircRUNX1 expression in Nthy-ori 3-1 cells and PTC cell lines (KTC, TPC-1, B-CPAP, K1 and IHH-4) was evaluated by qRT-PCR. **e** Schematic illustration showing RUNX1 exons 5–6 circularization to form circRUNX1 (black arrow). The presence of circRUNX1 was validated by RT–PCR and Sanger sequencing, and the head-to-tail splicing site of circRUNX1 is represented by the red arrow. **f** Agarose gel electrophoresis showed the presence of circRUNX1 in TPC-1 and B-CPAP cell lines by RT–PCR. Divergent primers amplified circRUNX1 from cDNA but not from genomic DNA. GAPDH was used as a negative control. **g** The expression of circRUNX1 and RUNX1 mRNA in TPC-1 and B-CPAP cells treated with or without RNase R by RT-qPCR. **h** RNA fluorescence in situ hybridization (FISH) revealed that circRUNX1 was predominantly localized in the cytoplasm. Nuclei were stained with DAPI, and circRUNX1 probes were labeled with Alexa Fluor 555. Scale bar = 20 μm. Data represent the mean ± SD from three independent experiments (**d** and **g**) (*P < 0.05 by Student’s *t*-test).

To generate a circRNA profiling database, three pairs of representative PTC and paracarcinoma tissues were selected for circular RNA microarray analysis. The clinical characteristics of the sequenced specimens are provided in Additional file 1: Supplementary Table S1. Differential expression patterns of circRNAs were identified by fold-change filtering, and a heatmap of the top 100 most upregulated circRNAs was generated (Fig. 1a). To validate the RNA microarray sequencing results, we performed qRT-PCR analysis to identify the 10 most differentially expressed circRNAs (Additional file 2: Supplementary Fig. S1A), and the results indicated that hsa_circ_0002360 showed the highest upregulation in the PTC tissues compared with the control tissues. Therefore, we selected hsa_circ_0002360 for further analysis. This circRNA is formed by the circularization of exons 5–6 of the RUNX1 gene (hereafter referred to as circRUNX1), and no further studies have been reported on this circRNA in the field of thyroid cancer research.

### CircRUNX1 is upregulated in human papillary thyroid cancer samples and predominantly localized in the cytoplasm

To further verify the RNA sequencing results, circRUNX1 expression was detected in 52 paired PTC tissues and adjacent tissues using qRT-PCR (Fig. 1b), and the clinicopathological characteristics are shown in Additional file 1: Supplementary Table S6. The results showed that the circRUNX1 expression level was significantly higher in patients with larger tumor size, advanced TNM stage, extrathyroidal extension and lymph node metastasis, which indicated that circRUNX1 may play an important role in the progression of PTC and might therefore be considered a novel prognostic biomarker. The abundant expression of cytoplasmic circRUNX1 in PTC by RNA FISH analysis further verified this point (Fig. 1c). Consistent with the results of the clinical samples, the expression of circRUNX1 was higher in multiple PTC cell lines than normal thyroid cells (Nthy-ori 3-1), and TPC-1 and B-CPAP cells were selected for further investigation (Fig. 1D). Sanger sequencing was conducted to confirm the predicted head-to-tail splicing junction in the RT-qPCR product of circRUNX1 identified by its expected size (2867 base pairs (bp)) using well-designed divergent primers (Fig. 1e). To rule out the possibility that head-to-tail splicing may be produced by genomic rearrangement or trans-splicing, RNase R was used to treat RUNX1 and circRUNX1 mRNA. CircRUNX1 showed strong tolerance to the action of RNase R (Fig. 1f) and was detected only from the cDNA of TPC-1 and B-CPAP cells using convergent and divergent primers, while RUNX1 was detected from both cDNA and gDNA (Fig. 1g). Furthermore, FISH assays demonstrated that circRUNX1 was mainly located in the cytoplasm (Fig. 1h).

### CircRUNX1 silencing inhibits the migration, invasion and proliferation of PTC cells

**Fig. 2:**
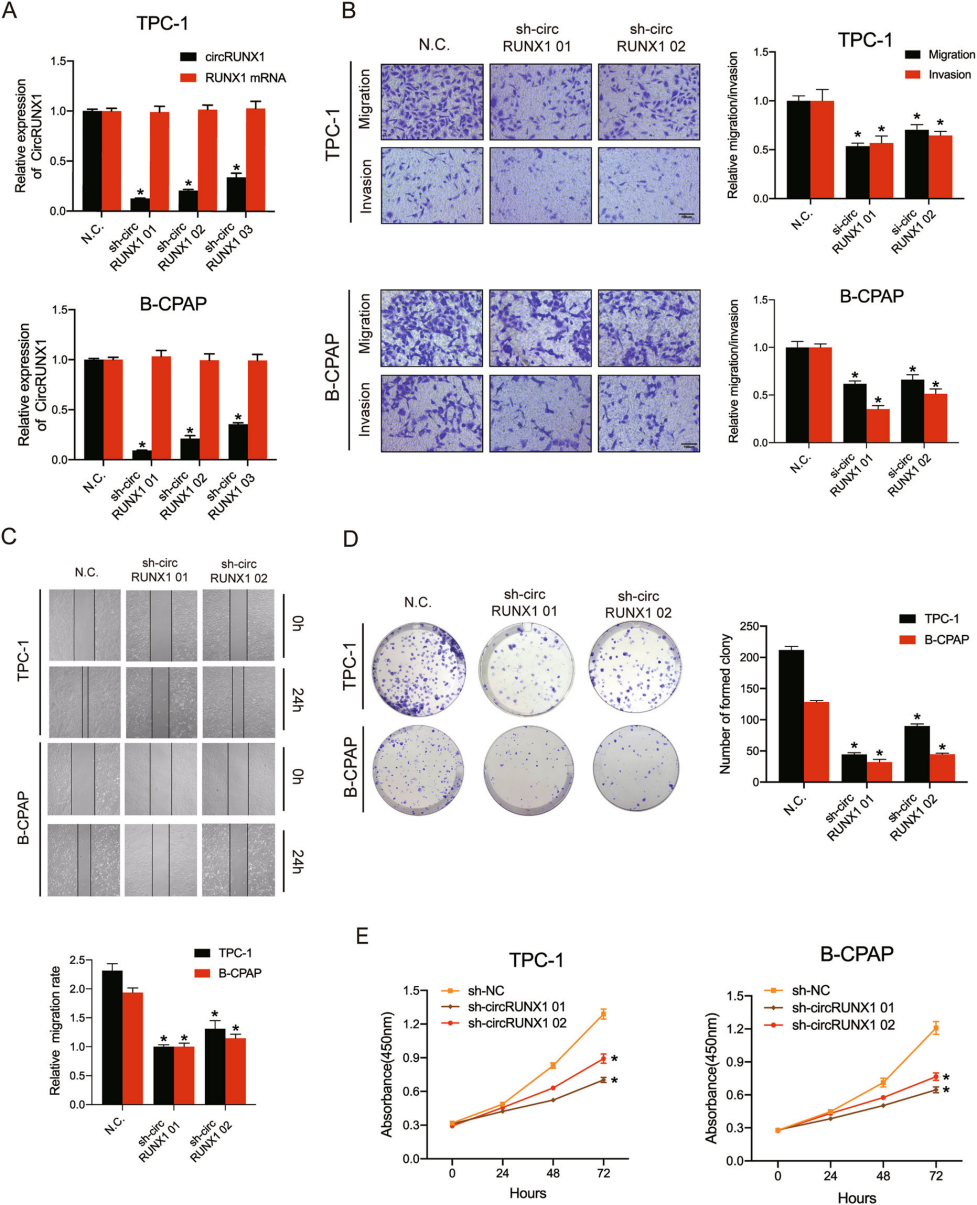
CircRUNX1 silencing inhibits the migration, invasion and proliferation of PTC cells. **a** TPC-1 and B-CPAP cells were stably transfected with circRUNX1 short hairpin RNAs or the vector plasmid, and the expression levels of circRUNX1 and RUNX1 mRNA were detected by real-time PCR. **b** Transwell migration and Matrigel invasion assays were conducted to evaluate the migration and invasion abilities of TPC-1 and B-CPAP cells transfected with sh-circRUNX1 or vector. Scale bar = 100 μm. **c** The effect of sh-circRUNX1 on migration was evaluated by the wound-healing assay in TPC-1 and B-CPAP cells. **d** ShRNA-mediated circRUNX1 knockdown significantly suppresses cell growth in a colony formation assay. **e** Proliferation of TPC-1 and B-CPAP cells transfected with sh-circRUNX1 was measured by CCK-8 assay. Data represent the mean ± SD from three independent experiments (**a**–**e**) (*P < 0.05 by Student’s *t*-test).

To explore the function of circRUNX1 in PTC cells, 3 well-designed circRUNX1 small hairpin RNAs (shRNAs) that targeted the junction sites of circRUNX1 and could stably knock down the expression of circRUNX1 in most cells were transfected into TPC-1 and B-CPAP cells. On the other side, circRUNX1 stably overexpressing cell lines were established via transfecting with circRUNX1 overexpression plasmid. The silencing and overexpression efficiency was detected by qRT-PCR, and circRUNX1 expression was significantly decreased to varying degrees by the shRNAs and overexpressed by circRUNX1 vector, while RUNX1 mRNA did not change (Fig. 2a and Additional file 3: Supplementary Fig. S2A). Among the shRNAs, sh-circRUNX1 01 showed the best knockdown efficiency. Knockdown of circRUNX1 markedly suppressed the migration and invasion abilities of PTC cell lines in Transwell migration and Matrigel invasion assays and the wound healing assay (Fig. 2b, c). The proliferative capability of PTC cells was then evaluated by a colony formation assay and a proliferation assay, which showed a critical impact on cell proliferation upon cell transfection with circRUNX1 shRNA compared to controls (Fig. 2d, e). In contrast, high levels of circRUNX1 promoted the proliferation, migration and invasion of papillary thyroid cancer cells (Additional file 3: Supplementary Fig. S2b–e). Collectively, these data indicate that circRUNX1 is involved in PTC cell proliferation and motility in vitro.

### CircRUNX1 acts as a sponge for miR-296-3p in vitro

**Fig. 3:**
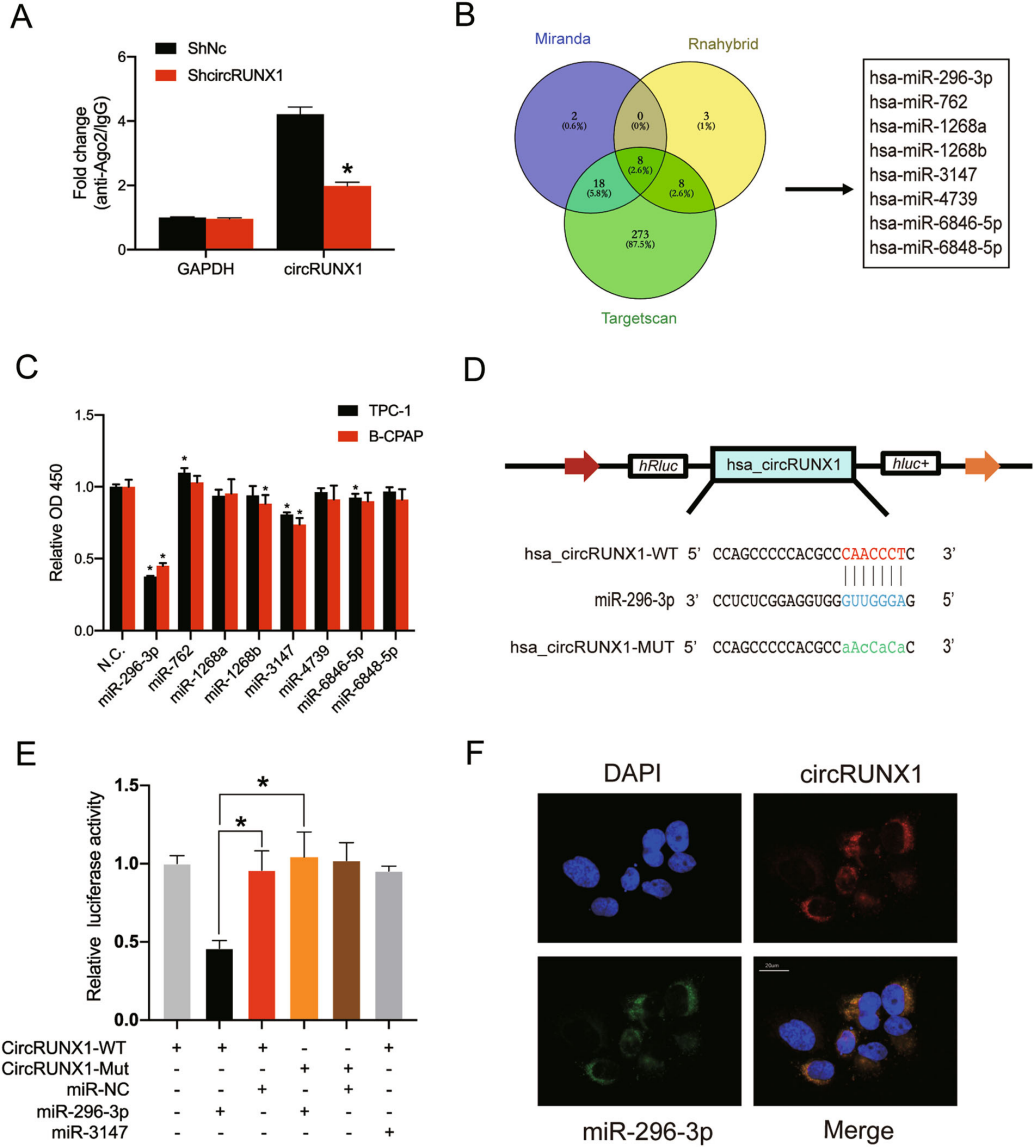
CircRUNX1 acts as a sponge for miR-296-3p in PTC cells. **a** Ago2 RNA immunoprecipitation (RIP) assay was performed to detect circRUNX1 levels in HEK-293T cells transfected with Ago2. **b** Schematic diagram showing the microRNAs predicted to bind to circRUNX1 through biological information analysis. The intersection of three databases (RNAhybrid, miRanda, and TargetScan) is displayed. **c** PTC cell lines were transfected with these potential microRNA mimics, and forty-eight hours later, cells were cultured in 96-well plates for another five days. The relative optical density (OD 450) was determined using the CCK-8 assay. **d** Schematic illustration of the binding sequence between circRUNX1 and miR-296-3p. Mutated nucleotides of the circRUNX1 3′UTR are represented in lowercase letters. **e** HEK-293T cells were co-transfected with miR-296-3p, miR-3147 mimics or mimics NC and wild-type or mutated circRUNX1 luciferase reporter and subjected to the luciferase assay. **f** FISH revealed colocalization between miR-296-3p and circRUNX1 in B-CPAP cells. CircRUNX1 probes were labeled with Alexa Fluor 555. MiR-296-3p probes were labeled with Alexa Fluor 488. Nuclei were stained with DAPI; Scale bar = 20 μm. Data represent the mean ± SD from three independent experiments (**a** and **c**) (*P < 0.05 by Student’s *t*-test).

One of the biological functions of circRNAs is to act as miRNA sponges and further influence the mRNA targeted by the corresponding miRNA^14,15,23^. Given that circRUNX1 is enriched in the cytoplasm, we speculated that circRUNX1 may act as a miRNA sponge to play an important role in the biological behavior of PTC. To confirm our hypothesis, HEK-293T cells were transfected with the AGO2 plasmid or control vector for RNA immunoprecipitation with an antibody targeting AGO2. Endogenous circRUNX1 was less enriched in the sh-circRUNX1 stably transfected group than in the control group, as demonstrated by qRT-PCR, suggesting that circRUNX1 interacts and binds with miRNAs through AGO2 protein (Fig. 3a). Then, three bioinformatics databases (miRanda, TargetScan and RNAhybrid) were used to predict the potential target miRNAs, and 8 miRNAs were identified from the overlap between the databases (Fig. 3b). Then, we performed a CCK-8 assay with these miRNAs, miR-296-3p and miR-3147 showed a significant impact on cell proliferation in PTC cells (Fig. 3c). Bioinformatic analysis revealed that circRUNX1 contains a potential target site of miR-296-3p (Fig. 3d) and miR-3147. To further verify the interactions, HEK-293T cells were transfected with miR-296-3p or miR-3147 mimics. Compared with the control, miR-296-3p mimics caused a significant decrease in the luciferase activity with the circRUNX1-WT reporter, while miR-3147 showed a weaker binding ability to circRUNX1 (Fig. 3e). Furthermore, the RNA FISH assay indicated a high degree of colocalization between circRUNX1 and miR-296-3p in B-CPAP cells (Fig. 3f). These results suggested that miR-296-3p can be sponged by circRUNX1.

### MiR-296-3p suppresses PTC cell proliferation and motility

**Fig. 4:**
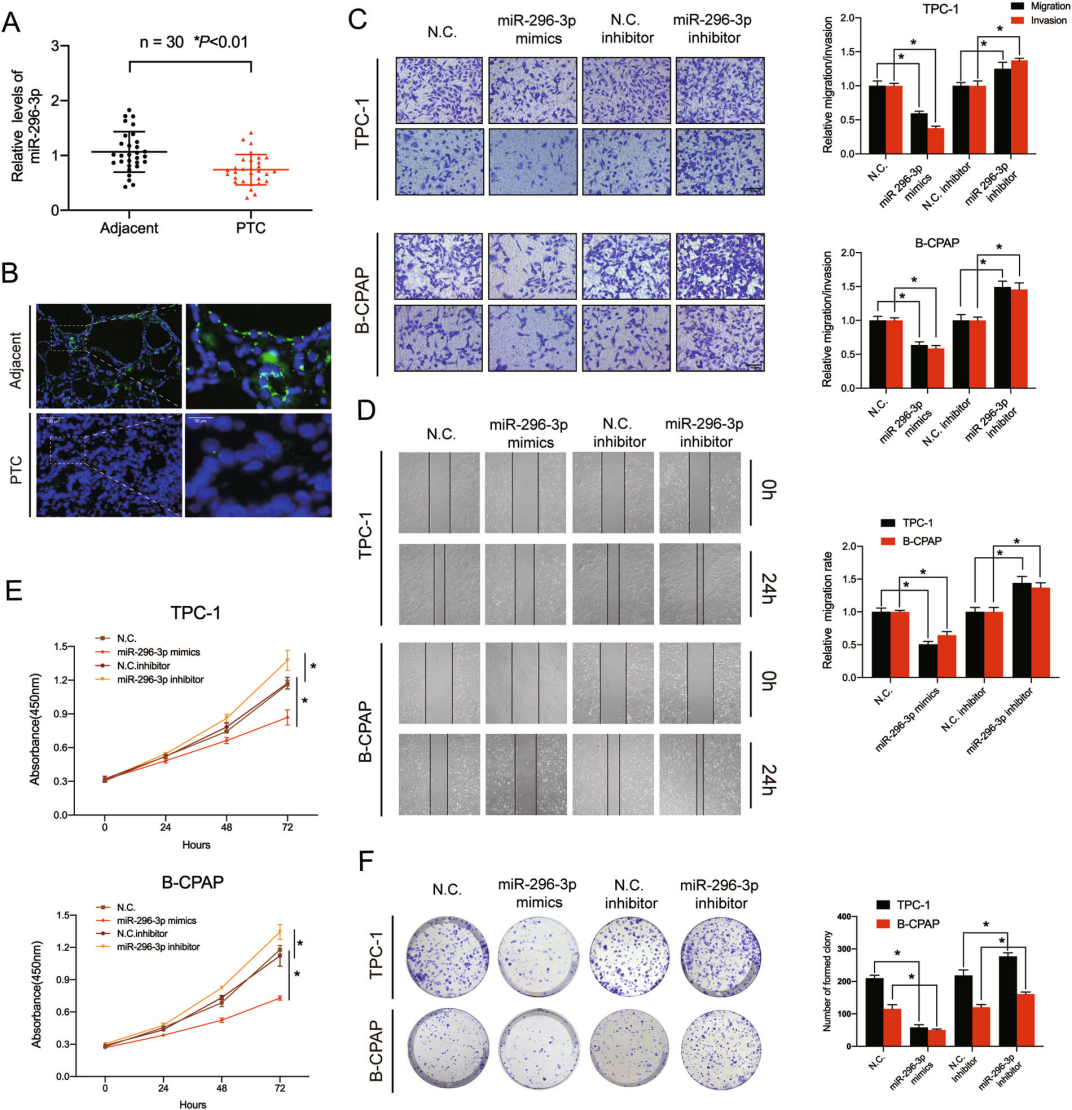
MiR-296-3p suppresses PTC cell proliferation, migration, and invasion. **a** Low levels of miR-296-3p were expressed in human PTC tissues compared with adjacent normal tissues (*n* = 30) (*P < 0.01, Paired Student’s *t*-test). **b** FISH assay indicated that miR-296-3p expression was lower in PTC than in paracarcinoma tissues. Representative images are shown. Scale bars = 100 μm or 50 μm. **c** PTC cell lines were transfected with miR-296-3p mimics or inhibitor, and cell migration and invasion were evaluated after 48 h. Scale bar = 100 μm. **d** Proliferation ability of transfected PTC cells was evaluated by the wound-healing assay. **e** Downregulation of miR-296-3p stimulates cell growth, and overexpression of miR-296-3p suppresses cell growth in TPC-1 and B-CPAP cells. **f** Representative images of the colony formation assay showing changes in the proliferation capacity of stable PTC cells. Data represent the mean ± SD from three independent experiments (**c**–**f**) (*P < 0.05 by Student’s *t*-test).

The complex role of miR-296-3p as an oncogene or tumor suppressor has been described in several cancer types but has rarely been described in PTC^24–27^. Here, we found that the expression of miR-296-3p was significantly decreased in PTC tissues compared with adjacent tissues using qRT-PCR (Fig. 4a), and RNA FISH analysis revealed lower expression of miR-296-3p in tumor tissues, which had the opposite trend to circRUNX1 expression mentioned in the above article (Fig. 4b). The expression of miR-296-3p was also found to be significantly downregulated in PTC cell lines (Additional file 4: Supplementary Fig. S3A). Considering that circRUNX1 is able to sponge miR-296-3p, we then evaluated the role of miR-296-3p in PTC by transfecting TPC-1 and B-CPAP cells with the miR-296-3p mimics or inhibitor. The transfection efficiency was detected by qRT-PCR (Additional file 4: Supplementary Fig. S3B). As shown in Fig. 3c, miR-296-3p overexpression decreased the migration and invasion ability of PTC cells, while miR-296-3p downregulation promoted this process in both TPC-1 and B-CPAP cells (Fig. 4c). The wound-healing assay further confirmed the decreased migration of PTC cells transfected with miR-296-3p mimics and the increased migration in those transfected with miR-296-3p mimics (Fig. 4d). Furthermore, the CCK-8 assay along with plate colony formation assays revealed that higher miR-296-3p levels in cells compromised cell proliferative capacity, while their miR-296-3p inhibition led to an increase proliferative capacity compared with that in the NC group (Fig. 4e, f). Overall, these data indicate that miR-296-3p functions as a suppressor of the migration, invasion and proliferation of PTC cells in vitro.

### Silencing miR-296-3p reverses the sh-circRUNX1-induced antitumor effects in PTC cells

**Fig. 5:**
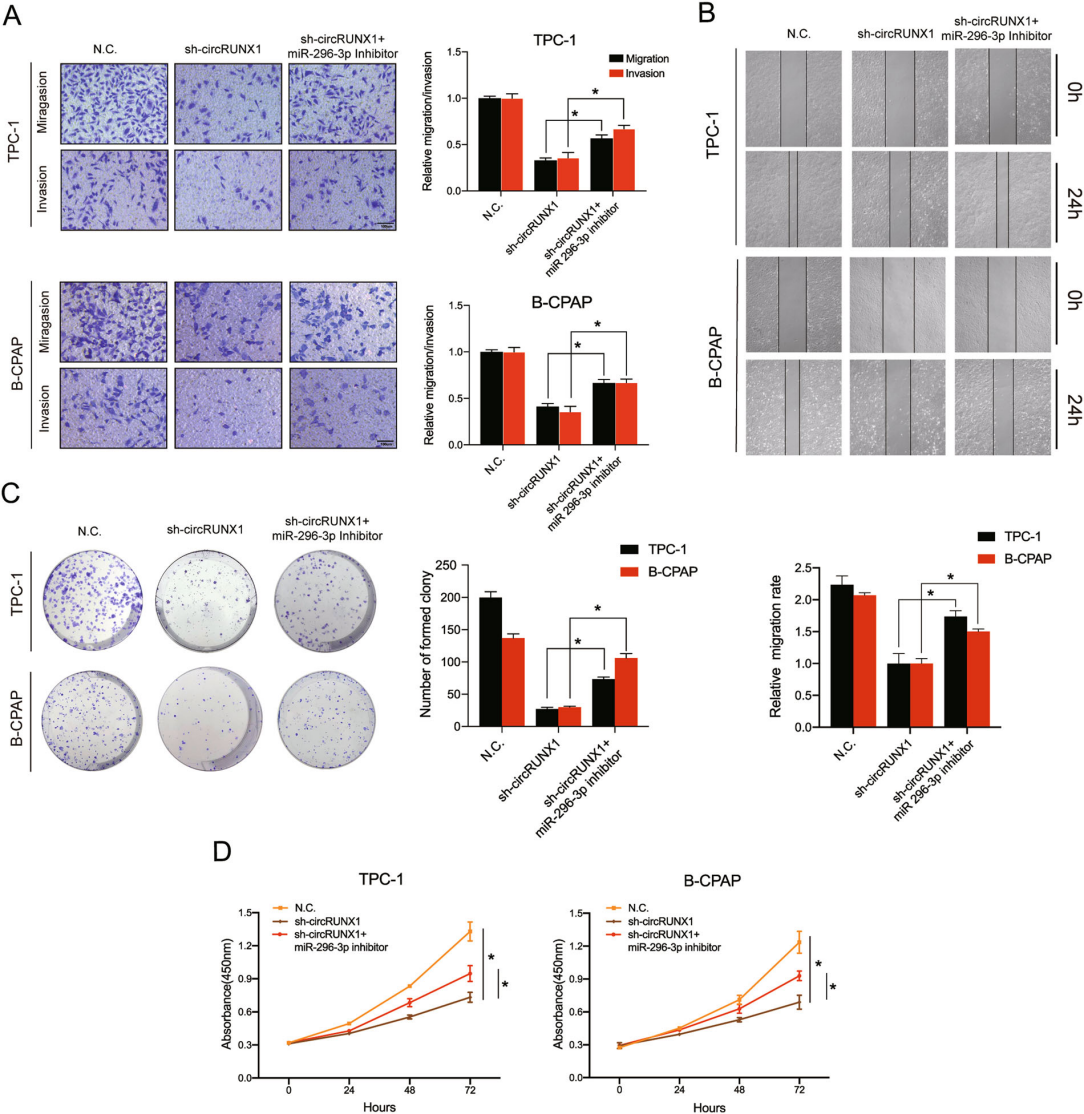
Knockdown of miR-296-3p reverses the sh-circRUNX1-induced antitumor effects in PTC cells. **a** PTC cell lines were cotransfected with stable sh-NC and inhibitor-NC or sh-circRUNX1 and inhibitor-NC or sh-circRUNX1 and miR-296-3p inhibitor. The migration and invasion potential was evaluated by Transwell migration and Matrigel invasion assays. Scale bars = 100 μm. **b** Representative images showing the reversion of the migration ability upon miR-296-3p downregulation by wound-healing assay. **c** Colony formation assay exhibiting changes in the proliferation capacity of stable sh-circRUNX1 or sh-NC PTC cells transfected with or without the miR-296-3p inhibitor. **d** The CCK-8 assay revealed that the effect of circRUNX1 knockdown on cell growth was abrogated by miR-296-3p downregulation. Data represent the mean ± SD from three independent experiments (**a**–**d**) (*P < 0.05 by Student’s *t*-test).

As we hypothesized that circRUNX1 promotes PTC progression mainly by sponging miR-296-3p, it was important to determine whether miR-296-3p can reverse the sh-circRUNX1 effect on PTC cells. Hence, several rescue experiments were performed by stably cotransfecting sh-circRUNX1 and sh-NC TPC-1 and B-CPAP cells with miR-296-3p inhibitor or control vector. Knockdown of both miR-296-3p and circRUNX1 could partly rescue the loss of motility abilities of sh-circRUNX1 PTC cell lines compared with the sh-NC group, as demonstrated by Transwell migration and Matrigel invasion assays (Fig. 5a). The wound-healing assay confirmed this finding (Fig. 5b). In addition, plate colony formation and CCK-8 assays indicated that the inhibition of cell proliferation ability was reversed by the exogenous downregulation of miR-296-3p expression (Fig. 5c, d).

### DDHD2 is a direct target of miR-296-3p and is considered an oncogene in PTC

**Fig. 6:**
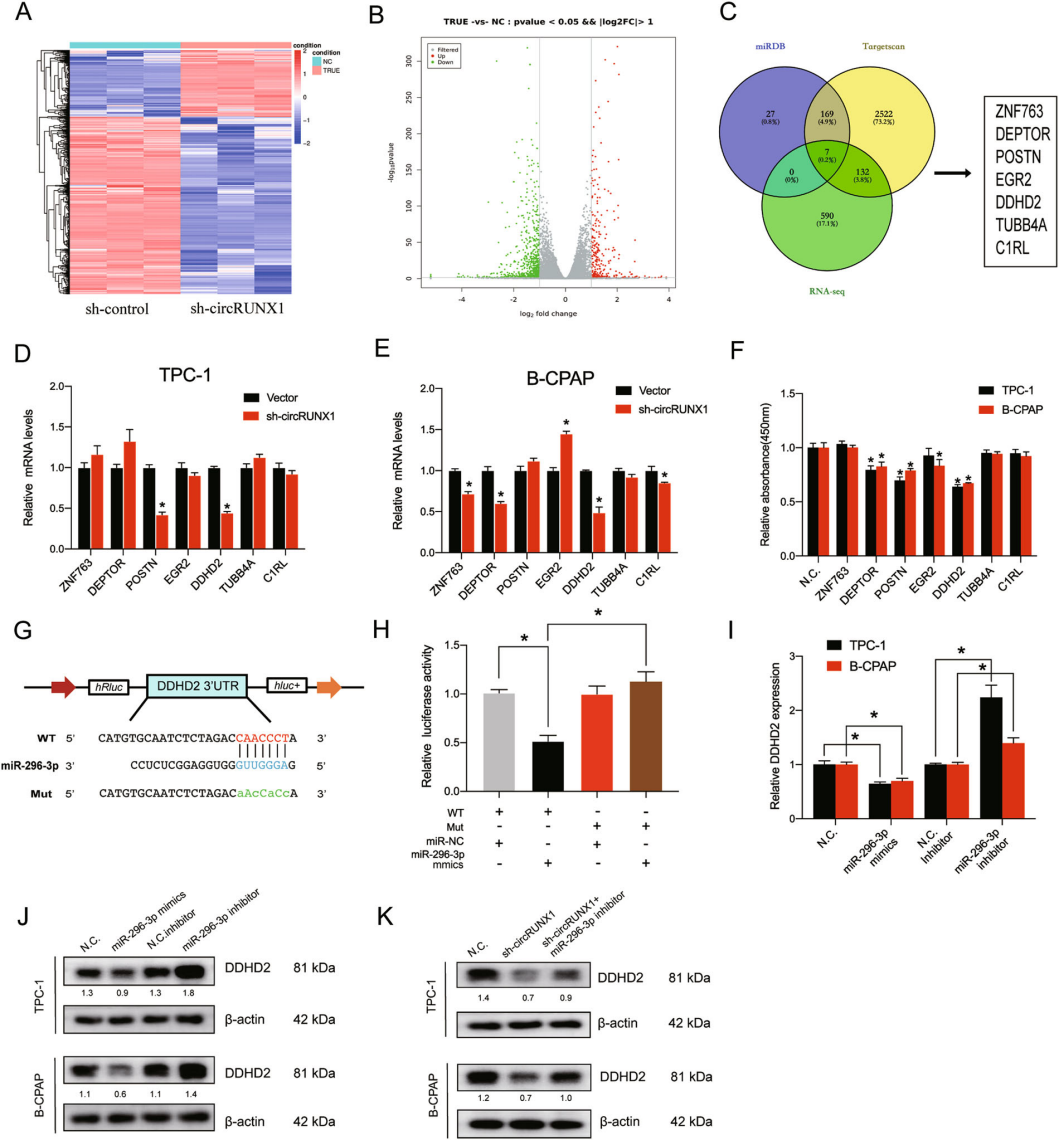
DDHD2 is a direct target of miR-296-3p. **a**, **b** Clustered heatmap and volcano plots of significantly differentially expressed mRNAs based on TPC-1 cells transfected with sh-control or sh-circRUNX1. Samples included three replicates. **c** Schematic diagram showing the intersection of target gene analyses of miR-296-3p from the TargetScan and miRDB databases and the downregulated mRNAs in sh-circRUNX1-silenced cells. **d**, **e** The histograms show the relative mRNA expression of 7 selected genes in TPC-1 and B-CPAP cells transfected with NC or sh-circRUNX1 by qRT-PCR. (*P < 0.01 by Student’s *t*-test). **f** Both TPC-1 and B-CPAP cells were transfected with siRNAs against these potential genes, and forty-eight hours later, cells were cultured in 96-well plates for another five days. The relative optical density (OD 450) was determined using the CCK-8 assay. **g** Schematic illustration of the binding sequence between miR-296-3p and DDHD2. Mutated nucleotides of the DDHD2 3′UTR are represented in lowercase letters. **h** HEK-293T cells were transfected with miR-296-3p mimics or NC and wild-type or mutated DDHD2 3′-UTR reporter and subjected to the luciferase assay. **i** The mRNA levels of DDHD2 in PTC cells transfected with miR-296-3p mimics (or NC mimics) or miR-296-3p inhibitor (or NC inhibitor) were evaluated by qRT-PCR. **j** PTC cells were stably transfected with sh-circRUNX1 or sh-NC and treated with miR-296-3p inhibitor or inhibitor-NC. DDHD2 expression was detected by Western blotting at the protein level. Densitometry was calculated using ImageJ and relative densitometry ratio (compared with corresponding beta-actin) was displayed below the band. **k** The protein expression of DDHD2 was evaluated by Western blotting in PTC cells transfected with sh-circRUNX1 or co-transfected with sh-circRUNX1 and miR-296-3p inhibitor. Densitometry was calculated using ImageJ and relative densitometry ratio (compared with corresponding beta-actin) was displayed below the band. Data represent the mean ± SD from three independent experiments (**d**–**g**, **i**) (*P < 0.05 or as indicated by Student’s *t*-test).

The function of circRUNX1 acting as a potent miR 296-3p miRNA sponge prompted us to focus on the target genes. The transcriptome of B-CPAP cells with stable circRUNX1 silencing and control cells was sequenced by RNA-seq to identify the significantly differentially expressed genes, which identified 729 downregulated genes (Fig. 6a, b). The corresponding KEGG and GO analyses and Circos plots are shown in Additional file 5: Supplementary Fig. S4A–D. The possible targets of miR-296-3p were next predicted by bioinformatic analysis using TargetScan and miRDB, and seven genes (ZNF763, DEPTOR, POSTN, EGR2, DDHD2 and TUBB4A and C1RL) were selected by overlapping the predicted results of potential miR-296-3p targets and RNA-seq data (Fig. 6c). RT-qPCR was further conducted to confirm this result, which showed that DDHD domain containing 2 (DDHD2) gene was downregulated by circRUNX1 in both TPC-1 and B-CPAP cells (Fig. 6d, e). Moreover, DDHD2 had the most promotion effect on cell proliferation in PTC cells (Fig. 6f) and was therefore selected for further investigation. Bioinformatic analysis using miRNA target prediction software revealed that miR-296-3p could potentially target DDHD2 (Fig. 6g), and the luciferase activity of HEK-293T cells transfected with miR-296-3p mimics showed an decrease in the luciferase activity of the DDHD2-WT 3′-UTR reporter but not in that of the DDHD2-MUT 3′-UTR reporter (Fig. 6h). Furthermore, RT-qPCR and western blot analysis revealed that DDHD2 expression was inversely regulated by the miR-296-3p mimics or inhibitor in PTC cells, suggesting that DDHD2 is a true miR-296-3p target (Fig. 6i, j). The decrease in DDHD2 expression in sh-circRUNX1 stably transfected PTC cells was inversely regulated by miR-296-3p inhibitor in PTC cells at the protein level (Fig. 6k).

DDHD2 has been reported to act as an oncogene in various cancers, but its role in PTC remains unknown^28–30^. To determine whether endogenous DDHD2 expression is closely related to PTC pathogenesis, PTC cells were then transfected with si-DDHD2 to evaluate its function in vivo. The transfection efficiency was verified by qRT-PCR (Additional file 6: Supplementary Fig. S5A). Lower levels of DDHD2 led to the inhibition of migration and invasion, as demonstrated by Transwell migration assays, Matrigel invasion assays and wound-healing assays (Additional file 6: Supplementary Fig. S5B, C). On the other hand, PTC cells with downregulated DDHD2 expression showed a lower growth rate and decreased colony formation compared with controls (Additional file 6: Supplementary Figures S5D, E). Taken together, our results indicate that miR-296-3p overexpression may inhibit PTC progression via DDHD2.

### CircRUNX1 promotes PTC progression via DDHD2

**Fig. 7:**
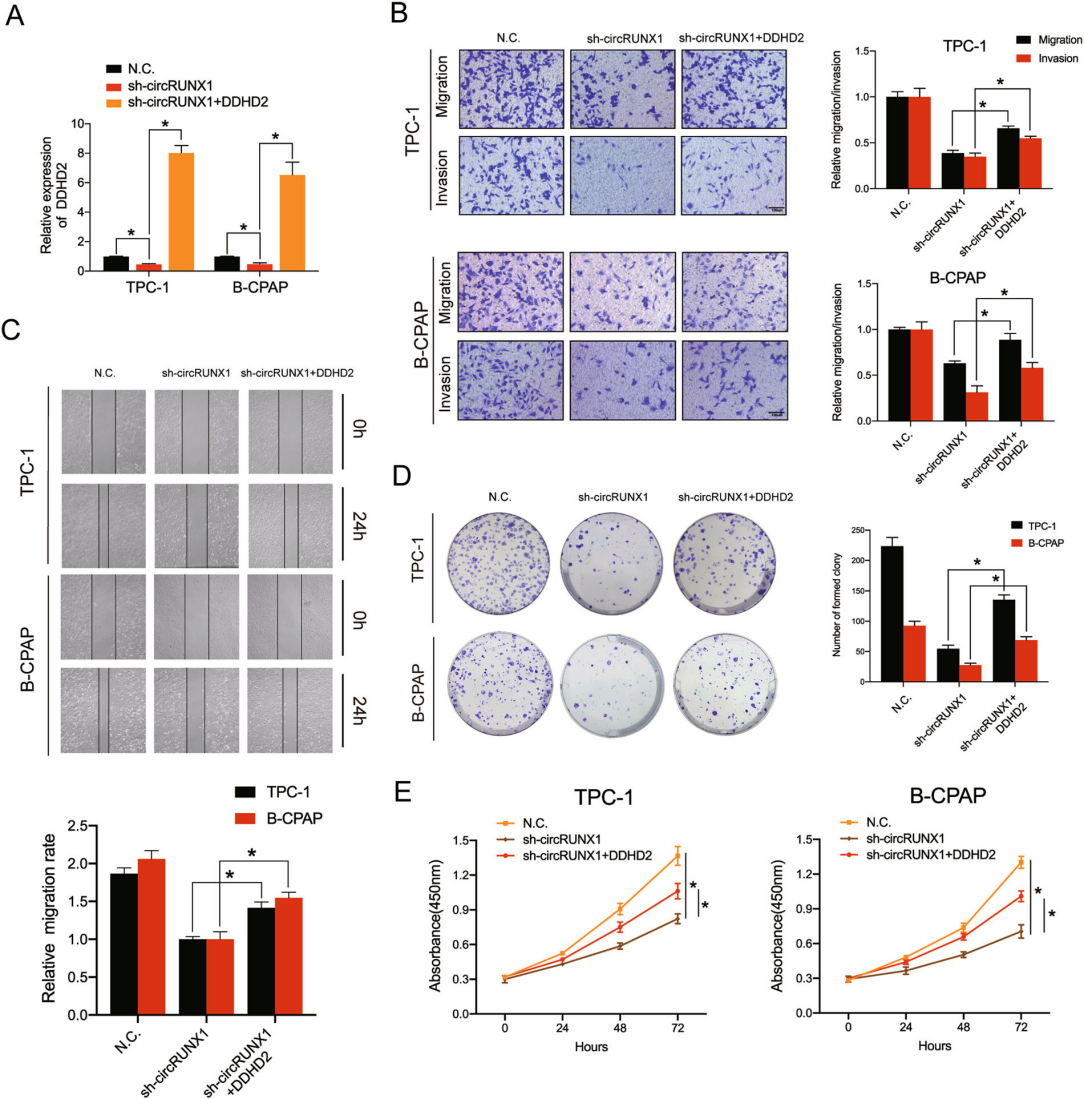
DDHD2 mediates the tumor-suppressive function of circRUNX1 in PTC cells in vitro. **a** qRT-PCR was conducted to evaluate DDHD2 mRNA expression in PTC cells transfected with sh-NC or sh-circRUNX1 or cotransfected with sh-circRUNX1 and the DDHD2 overexpression plasmid. **b** The decreased migration and invasion potential of circRUNX1 knockdown cells was abrogated by DDHD2 overexpression in cells cotransfected with sh-circRUNX1 and DDHD2, as demonstrated by Transwell migration and Matrigel invasion assays. Scale bars = 100 μm. **c** Representative images showing the reversion of the effect on migration ability by DDHD2 overexpression, as demonstrated by the wound-healing assay. **d** The colony formation assay showing changes in the proliferative capacity of stable sh-circRUNX1 or sh-NC PTC cells with or without DDHD2 overexpression. **e** The CCK-8 assay revealed that the effect of circRUNX1 silencing on cell growth was abrogated by DDHD2 overexpression. Data represent the mean ± SD from three independent experiments (**a**–**e**) (*P < 0.05 by Student’s *t*-test).

A DDHD2 overexpression plasmid was constructed and transfected into circRUNX1-stably silenced TPC-1 and B-CPAP cells to further investigate whether circRUNX1 influences PTC progression by targeting DDHD2. The overexpression efficiency was confirmed by RT-qPCR and Western blot (Fig. 7a and Additional file 6: Supplementary Fig. S5F). Then, the migration and invasion ability was evaluated to explore the impact of DDHD2 overexpression on circRUNX1-deficient PTC cells, which proved that high levels of DDHD2 could clearly rescue the downregulation of motility in circRUNX1-deficient cells. (Fig. 7b, c). Additionally, the colony-forming ability and proliferative capacity of the cells evaluated by the CCK-8 assay could be improved by overexpressing DDHD2 compared with that of the sh-circRUNX1-treated cells (Fig. 7d, e). Altogether, these findings indicate that circRUNX1 participates in PTC progression mainly by targeting DDHD2.

### CircRUNX1 acts as a sponge of miR-296-3p to promote tumorigenesis in vivo

**Fig. 8:**
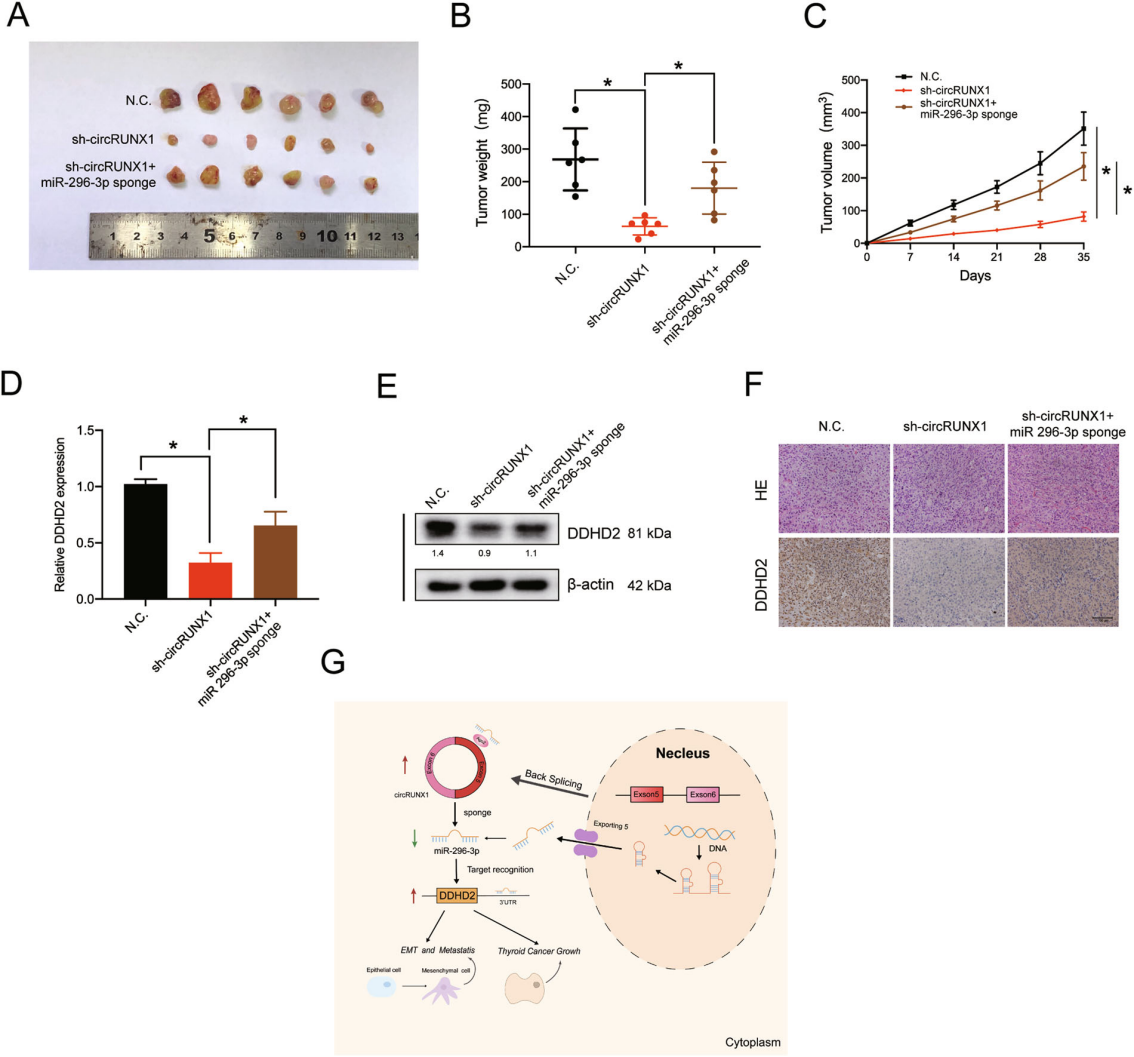
CircRUNX1 acts as a sponge of miR-296-3p to promote tumorigenesis in vivo. **a** Nude mice were subcutaneously injected with 5 × 10^6^ stable control cells or cells transfected with circRUNX1 shRNA or cotransfected with circRUNX1 shRNA and miR-296-3p inhibitor. After 5 weeks, tumors were dissected and imaged. **b** Average tumor weight was calculated when mice were euthanized. Data represent the mean ± SEM (*n* = 6 each group). **c** Tumor volumes (ab ^2^/2) were recorded every seven days after mice were injected with stable PTC cells. Data represent the mean ± SEM (*n* = 6 each group). *P < 0.05. **d** qRT-PCR analysis of DDHD2 expression in tumors from xenograft mice. (*n* = 6 in each group). **e** Western blot analysis of DDHD2 in tumors from different groups. Densitometry was calculated using ImageJ and relative densitometry ratio (compared with corresponding beta-actin) was displayed below the band. **f** H&E and IHC staining revealed the tumor structure and relative protein levels of DDHD2 in tumors. Scale bars, 100 μm. **g** Schematic illustration of the circRUNX1/miR-296-3p/DDHD2 axis. *P < 0.05 by Student’s *t*-test.

To determine whether circRUNX1 and miR-296-3p play a role in PTC progression in vivo, a xenograft tumor model was established. TPC-1 cells that were stably transfected with NC or sh-circRUNX1 or cotransfected with sh-circRUNX1 and the miR-296-3p sponge were injected separately into the nude mice. As shown in Fig. 8a, b, the circRUNX1 knockdown group had lower proliferation rate compared to the NC group, whereas cells silenced for both circRUNX1 and miR-296-3p partially reversed the decrease in proliferation. The final tumor weights of the three groups also showed the same difference. In additon, the volume of TPC-1-derived tumors were decreased by circRUNX1 knockdown in vivo (Fig. 8c), Total RNA and protein were then extracted from the tumors to investigate the in vivo correlation of circRUNX1, miR-296-3p and DDHD2. CircRUNX1 silencing markedly reduced DDHD2 expression at both the mRNA and protein levels, which could be reversed by the miR-296-3p sponge (Fig. 8d, e). Accordingly, the mean immunopositive area for DDHD2 was decreased under the influence of sh-circRUNX1, as determined by immunohistochemistry, and the inhibition of miR-296-3p again counteracted this change (Fig. 8f). These results indicate that circRUNX1 may play an important role in promoting the proliferation of PTC in vivo via miR-296-3p (Fig. 8g).

## Discussion

As the most common endocrine malignancy at present, the incidence of PTC continues to rise worldwide. Although the prognosis of most PTC patients tends to be satisfactory with standard treatment, some patients still show rapid tumor progression, multiple distant metastases, and even death^31–33^ caused by highly heterogeneous biological behaviors and morphological features^34^. It is important to investigate the underlying mechanisms of PTC, which may be of great help to the screening, diagnosis and treatment of PTC in the future.

CircRNAs have attracted great attention in recent years as a type of noncoding RNA that is widespread, tissue specific and conserved in mammalian cells. Recently, many circRNAs were found to play important roles in the progression and metastasis of lung cancer, bladder cancer and gastric cancer^35–37^, while little is known about their functions in the development and progression of PTC. In the present study, we focused on a specific circular RNA, circRUNX1, as a key circRNA involved in PTC. CircRUNX1 is abundantly expressed in PTC and results in worse biological behavior at high levels, indicating its important role in PTC and prompting us to further explore its function in the progression and epithelial-mesenchymal transition of PTC.

Runx1 is a member of the runx gene family, which has rich and diverse functions and can play various roles according to the cell environments or differentiation pathways and is well known to be closely linked to blood diseases^38^. Recent studies have shown that Runx1, acting as an oncogene, is also closely related to the genesis and formation of a variety of cancers, such as ovarian cancer, esophageal cancer and breast cancer^39–41^. Additionally, it was demonstrated to contribute to the recurrence of thyroid cancer^42^. CircRUNX1, as a circRNA originating from RUNX1 and abundantly expressed in PTC, may be partly involved in this cancer-promoting process. Coincidentally, a unique study involving circRUNX1 showed that circRUNX1 is the most enriched circRNA in lung adenocarcinoma compared to paracarcinoma tissue by circular RNA microarray analysis^43^, which suggests that its tumor-promoting function may effective in a variety of tumors. In the present study, loss-of-function and xenograft experiments were conducted and revealed that knockdown of circRUNX1 inhibited the proliferation and motility of PTC cells in vitro and in vivo.

CeRNA plays an important role in the pathogenesis of cancers, including PTC, by forming an extensive ceRNA network, and circRNAs have been found to function as ceRNAs in many pathophysiological and physiological conditions by binding miRNAs^36,37,44^. In our study, miR-296-3p had a high binding capacity with circRUNX1, which was validated by qPCR, luciferase and FISH analyses and was shown to play a significant role in the progression and metastasis of thyroid tumors. Furthermore, the expression of DDHD2 (miR-296-3p target) was positively regulated by circRUNX1. In general, we identified a mechanism wherein circRUNX1 acts as a miR-296-3p sponge to, thereby promote the progression of PTC.

Several miRNAs have been found to be differentially expressed in PTC and play tumor suppressor or promotor roles in tumors^45–47^. However, the effect of miR-296-3p on the biological behavior of different tumors is not completely consistent. According to previous studies, miR-296-3p reduces the proliferation, metastasis and chemotherapeutic drug resistance of lung adenocarcinoma cells^48^ and inhibits nasopharyngeal carcinoma^49^, while miR-296-3p can promote metastasis of prostate cancer by targeting intercellular adhesion molecule 1^50^. Furthermore, miR-296-3p is involved in the proliferation and cell cycle of normal rat thyroid cells via a thyrotropin-CREB1-miRNA loop that acts synergistically^51^. In the present study, miR-296-3p expression was found to be significantly downregulated in PTC and related to the proliferation and metastasis of PTC. Subsequent analyses showed that miR-296-3p suppressed DDHD2 expression, a mechanism reported herein for the first time. DDHD2 was recently widely discovered as an oncogene in breast cancer that stimulates the growth of cancer cells^29,30^, and DDHD2 was verified for the first time to act as a tumor driver gene in PTC progression. A distinctive decrease in DDHD2 expression levels led to the critical downregulated growth, migration and invasion ability of PTC.

In conclusion, we propose that targeting the circRUNX1/miR-296-3p/DDHD2 axis is a potential strategy for the treatment of PTC. Although the carcinogenic effect of circRUNX1 in PTC was confirmed in this study, we cannot exclude that there may be other key circRNAs involved in the genesis and development of PTC. Therefore, we will continue to pay attention to this field and perform further studies.

## Methods

### Ethics

All animal experiments were approved by the Ethics Committee of Sir Run Run Shaw Hospital and carried out under the guidelines of the Guide for the Care and Use of Laboratory Animals of the China National Institutes of Health.

### Clinical specimens

Thyroid cancer and paracarcinoma tissue samples were collected from the surgical specimen archives of the Department of Head and Neck Surgery of Sir Run Run Shaw Hospital, Zhejiang, China between January 2019 and August 2019. All tissues were histologically characterized by pathologists in accordance with the criteria established by the World Health Organization and were stored in liquid nitrogen after surgery. The patients did not receive any chemotherapy or radiotherapy prior to surgical resection, and three typical papillary thyroid carcinomas with matching adjacent normal tissues were ultimately selected for circRNA sequencing and bioinformatic analysis. Written informed consent was obtained from each patient before the beginning of this study.

### Cell culture and treatment

The human thyroid follicular epithelial cell line Nthy-ori 3-1 and the human PTC cell lines KTC-1, B-CPAP, TPC-1, K1, IHH-4 and HEK-293T were kindly provided by Stem Cell Bank, Chinese Academy of Sciences. Authentication of these cell lines were conducted by Chinese Academy of Sciences by short tandem repeat (STR) markers, and no mycoplasma contamination was detected. HEK-293T cells were maintained in DMEM, while the PTC cell lines and Nthy-ori 3-1 cells were maintained in RPMI-1640 medium with 10% fetal bovine serum (Gibco, Grand Island, NY, USA), 100 U/mL streptomycin, and 100 U/mL penicillin (Invitrogen, Carlsbad, CA, USA). Additional MEM Non-Essential Amino Acids Solution (NEAA 100X) (Invitrogen, 11140050) was added to the B-CPAP cell line. All the cells were incubated in humidified conditions at 37 °C with 5% CO_2_ and were determined to be mycoplasma-free.

### Subcutaneous xenograft tumor models

Approximately 5 × 10^6^ TPC-1 cells were injected subcutaneously into nude mice (female, 4 weeks old) (*n* = 6 per group) using a method of randomization. The width and length of the tumor were measured by Vernier calipers every week for 5 weeks, and the tumor volume was calculated based on the formula: volume (mm^3^) = 0.5 × (length × width^2^). Mice were sacrificed 5 weeks after injection, and tumors were harvested and weighed for RNA and protein extraction. The remaining tissue was fixed in 4% paraformaldehyde for further use.

### CircRNA plasmid construction and stable transfection

Small interfering RNAs (siRNAs) were obtained from RiboBio (Guangzhou, China) and transfected into cells with Lipofectamine iMax (Invitrogen) following the manufacturer’s instructions. Human lentivirus-sh-circRUNX1, circRUNX1-overexpressing lentiviral plasmid,lentivirus-miR-296-3p sponge and the DDHD2-overexpressing lentiviral plasmid were purchased from HanBio (Shanghai, China). microRNA mimics and inhibitors were purchased from GenePharma (Shanghai, China). The transfection efficiency was verified by qRT-PCR. The sequences of shRNA, siRNA and microRNA mimics and inhibitor are listed in Additional file 1: Tables S3-S4.

### RNA extraction, RNase R treatment, and real-time qRT-PCR

Total RNA was isolated from PTC tissues and cell lines using TRIzol reagent (Invitrogen) following the manufacturer’s instructions. MicroRNAs were isolated using a miRNA Purification Kit (Cwbiotech, Jaingsu, China). For the RNase R treatment, 2 μg of total RNA was incubated for 15 min at 37 °C with or without 3 U/mg RNase R (Geneseed, Guangzhou, China). To detect RNA expression, quantitative real-time PCR (qRT-PCR) was performed with a script RT reagent kit (TaKaRa) and SYBR Premix Ex Taq II (TaKaRa) and the Roche LightCycler 480II PCR instrument (Basel, Switzerland) for circRNA and mRNA analyses, while the Mir-X miR First-Strand Synthesis Kit (TaKaRa) and SYBR Premix Ex Taq II (TaKaRa) were used for miRNA analyses according to the manufacturer’s protocols. All primers were acquired from Tsingke Biological (Hangzhou, China). GAPDH or U6 was used as an internal control for circRNA and mRNA analyses or miRNA analyses. The 2^-ΔΔCt^ method was applied to quantify the fold change in gene expression. The primers are listed in Additional file 1: Supplementary Table S2.

### Nucleic acid electrophoresis

Given the different sizes of circRUNX1 and linear RUNX1 mRNA, cDNA (complementary DNA) and gDNA (genomic DNA) samples were separated using 2% agarose gel electrophoresis with TAE buffer. DNA was separated by electrophoresis at 130 V for 45 min. Super DNA Marker (CWBIO, Beijing, China) was used as a DNA marker. The bands were visualized by ultraviolet radiation.

### Fluorescence in situ hybridization (FISH)

Cy3-labeled circRUNX1 probes (specific for circRUNX1 and recognizing the junction 5′−3′) and fam-labeled miR-296-3p probes were designed and synthesized by RiboBio. Nuclei were counterstained with DAPI. The signals of the probes were determined with a Fluorescent In Situ Hybridization Kit (RiboBio) according to the manufacturer’s instructions. Images were acquired using an inverted fluorescence microscope (Nikon Eclipse Tisr, Tokyo, Japan). The probes are listed in Additional file 1: Supplementary Table S5.

### Prediction of miRNA targets of circRUNX1

We predicted the miRNA binding sites of circRUNX1 using the bioinformatics databases RNAhybrid (https://bibiserv.cebitec.unibielefeld.de/rnahybrid/), TargetScan (http://www.targetscan.org/) and miRanda (http://www.microrna.org/). Filtering restrictions were as follows: (i) Total score ≥ 155 and Total energy < −15 kcal/mol and (ii) Minimum free energy (MFE) ≤ −35 kcal/mol.

### Dual-luciferase reporter assay

Dual-luciferase reporter plasmids were purchased from Hanbio. HEK-293T cells were cultured in 24-well plates at a density of 3 × 10^4^ cells/well before transfection. Cells were then cotransfected with plasmid mixtures containing the RL reporter and FL reporter with or without the circRUNX1 3′-UTR (500 ng) and miR-296-3p mimics or negative control (NC) (10 nM final concentration) using Lipofectamine RNAiMAX (Invitrogen). The luciferase activity was measured with a Dual Luciferase Reporter Gene Assay Kit (Beyotime, Shanghai, China) after 48 h. For comparison, FLUC activity was normalized to RLUC activity to determine the ratio. Afterwards, the luciferase activity ratio of the miR-296-3p mimic group to the NC group was calculated and expressed as the fold-change.

### Transwell migration and Matrigel invasion assays

The migration and invasion assays were performed with Transwell plates (Millipore, Billerica, MA, USA, 8 μm). In brief, a density of 2 × 10^5^ cells were cultured in 200 μL medium without serum in the upper chamber, and 600 μL complete medium was added to the lower chamber for the migration assay. Additional Matrigel was used for the invasion assays according to the manufacturer’s protocols (BD Biosciences, Bedford, MA, USA). After incubation for 24 h, the cells were fixed with 4% paraformaldehyde and stained with 0.1% crystal violet. Migrated and invaded cells were quantified and counted in three random fields using an inverted light microscope (Zeiss, Primovert).

### CCK-8 assay

The proliferation ability of transfected cells was tested by a CCK-8 kit (Beyotime, Beijing, China) according to the manufacturer’s instructions. Cells were seeded in 96-well plates, and 10 µL CCK-8 reagent was added to each well at 0, 24, 48, and 72 h post treatment followed by incubation for 2 h. Optical density (OD) at 450 nm was measured using a spectrophotometer (Thermo Fisher Scientific, Vantaa, Finland). The experiments were repeated at least three times with three replicates each.

### Colony formation assay

Transfected TPC-1 and B-CPAP cells were plated into 12-well plates and cultured for 8 days at 37 °C. Then, colonies were fixed with 4% paraformaldehyde for 15 min and stained with 0.1% crystal violet. The colony formation numbers (>50 cells) were counted under a microscope, and the experiment was repeated three times independently.

### Wound-healing assay

Transfected TPC-1 and B-CPAP cells were cultured in six-well plates and subsequently scratched with a 200 μl pipette tip when the cells reached 80 to 90% confluence. After 24 h of cell culture, representative images of cell migration were captured by an inverted microscope, and the relative migration was calculated by the decreasing distance across the induced injury area normalized to the 0 h control.

### RNA immunoprecipitation

The Ago-RIP assay was conducted using the Magna RIP RNA-Binding Protein Immunoprecipitation Kit (Millipore, Bedford, MA). Stably transfected HEK-293 cells with sh-circRUNX1 or vector control were established first, and 1 × 10^7^ cells were lysed (200 μl) and incubated with 5 μg of control rabbit IgG or Argonaute-2 (AGO2) antibody (Abcam, MA, USA)-coated beads with rotation at 4 °C overnight. Total RNA was isolated to detect the circ-RUNX1 level and miRNA expression by qRT-PCR assay.

### Western blotting analysis and antibodies

The proteins in PTC cells were extracted using RIPA lysis buffer (P0013, Beyotime, China) containing protease inhibitor cocktails (FD1001, Fudebio, Hangzhou, China) on ice. Equal amounts of protein lysates were separated by SDS-PAGE gels at 120 V for 1.5 h and electroblotted onto polyvinylidene difluoride (PVDF) membranes (Amersham Bioscience, Piscataway, NJ) at 280 mA for 1.5 h. Membranes were blocked for 1 h with 5% skim milk powder in TBST in tris-buffered saline containing 0.1% Tween 20 and incubated overnight at 4 °C with specific primary antibodies. Then, the membranes were washed with TBST and incubated with an HRP-conjugated secondary antibody (FDM007 and FDR007, Fudebio, Hangzhou, China). The protein bands were visualized by chemiluminescence using a GE Amersham Imager 600 (GE, USA). Anti-beta actin and anti-DDHD2 antibodies were purchased from Proteintech (Chicago, USA). Primary antibody dilution buffer was purchased from Dalian Meilun Biotechnology Co., Ltd. (MB9881, Dalian, China).

### Immunohistochemistry (IHC)

IHC analysis was performed according to the manufacturer’s instructions. Briefly, tumor tissues were fixed with a formalin solution and dehydrated in ethanol, embedded in paraffin, and cut into 5 μm sections. Primary antibody against DDHD2 (Proteintech, Chicago, USA) was used and incubated with cells overnight at 4 °C. Then, the cells were washed in PBS, followed by incubation with secondary antibodies (Proteintech Group, Rosemount, IL, USA) for 1 h. Finally, the cells were washed in PBS, and immunofluorescence images were captured by an inverted microscope (Nikon Eclipse 80i, Tokyo, Japan).

### Statistical analyses

SPSS software version 20.0 (SPSS Inc., Armonk, NY, USA) was used for statistical analysis in this paper using unpaired Student’s *t*-test unless otherwise noted. Data were obtained from at least three independent experiments and are expressed as the means ± standard deviation (SD). A *p*-value <0.05 was considered statistically significant.

## Supplementary information

Supplementary figure legends

Additional file 1 Table S1-6

Additional file 2:Figure S1

Additional file 3:Figure S2

Additional file 4:Figure S3

Additional file 5:Figure S4

Additional file 6:Figure S5

## Data Availability

The datasets used and/or analyzed during the current study are available from the corresponding author on reasonable request.
