# Supplementary material for: Circular RNA circRUNX1 promotes papillary thyroid cancer progression and metastasis by sponging MiR-296-3p and regulating DDHD2 expression
Source: Cell Death Dis. 2021 Jan 21;12(1):112. doi: 10.1038/s41419-020-03350-8 (PMC7819993; doi:10.1038/s41419-020-03350-8)
Supplement: Supplementary file 1 — Supplementary figure legends [file 41419_2020_3350_MOESM1_ESM.docx]

**SUPPLEMENTARY FIGURE LEGENDS**

**Additional file 1:Table S1.** Detailed information of 3 patients is listed. **Table S2.**  Primers and RNA sequences used in this study. **Table S3.** ShRNA or siRNA sequences used in this study. **Table S4.** MiRNA mimics, and inhibitors sequences used in this study. **Table S5.** FISH probes sequences used in this study. **Table S6.** The clinical characteristics of the sequenced specimens.

**Additional file 2: Figure S1.** The expression of top 10 most high expressed circRNAs based on RNA deep sequencing results in 6 paired PTC tissues and adjacent tissues. Data represent the mean ± SD (*P < 0.05 by Student’s t-test).

**Additional file 3: Figure S2**. Overexpression of circRUNX1 increases the migration, invasion and proliferation of PTC cells in vitro. A TPC-1 and B-CPAP cells were stably transfected with circRUNX1 or the vector plasmid, and the expression levels of circRUNX1 and RUNX1 mRNA were detected by real-time PCR. B Migration and invasion abilities of TPC-1 and B-CPAP cells transfected with circRUNX1 or vector. Scale bar = 100 μm. C The effect of circRUNX1 overexpression on migration was evaluated by the wound-healing assay in TPC-1 and B-CPAP cells. D High levels of circRUNX1 accelerates cell growth in a colony formation assay. E Proliferation of TPC-1 and B-CPAP cells transfected with circRUNX1 was measured by CCK-8 assay. Data represent the mean ± SD from three independent experiments (A-E) (*P < 0.05 by Student’s t-test).

**Additional file 4: Figure S3.** The expression and transfection effects of miR-296-3p and DDHD2 in PTC cell lines. **A** The miR-296-3p expression in Nthy-ori 3-1 cells and PTC cell lines (KTC, TPC-1, and B-CPAP) was evaluated by qRT-PCR. **B** The miR-296-3p alteration of both TPC-1 and B-CPAP cells transfected with N.C. or miR-296-3p mimics or N.C. inhibitor or miR-296-3p inhibitor was determined by qRT-PCR. Data represent the mean ± SD (*P < 0.05 by Student’s t-test).

**Additional file 5: Figure S4.** Bioinformatic analysis of RNA sequencing data. **A** The comparison map of the differentially expressed genes and all genes at GO Level2 is as follows. **B** GO analysis of downregulated genes in sh-circRUNX1-treated cells is shown. **C** & **D** KEGG analysis of downregulated genes in sh-circRUNX1-treated cells.

**Additional file 6: Figure S5.** DDHD2 functions as a driver gene in PTC. **A** TPC-1 and B-CPAP cells were transfected with si-DDHD2. After 48 hours, the DDHD2 silencing efficiency was detected by qRT-PCR. **B** DDHD2 deficiency in PTC cells decreased the migration and invasion potential of both TPC-1 and B-CPAP cells, as demonstrated by Transwell migration and Matrigel invasion assays. Scale bars=100 μm. **C** Representative images showing the downregulation of the migration ability of DDHD2 knockdown cells by wound-healing assay. **D-E** Low levels of DDHD2 inhibited cell clone formation in TPC-1 and B-CPAP cells and strongly decreased the proliferation of cells. **F** Western blot was conducted to evaluate DDHD2 protien expression in PTC cells transfected with sh-NC or sh-circRUNX1 or cotransfected with sh-circRUNX1 and the DDHD2 overexpression plasmid. Data are from three independent experiments (mean ± SD) (**A-E**) (*P < 0.05 by Student’s t-test).
