# Supplementary material for: Circular RNA circRUNX1 promotes papillary thyroid cancer progression and metastasis by sponging MiR-296-3p and regulating DDHD2 expression
Source: Cell Death Dis. 2021 Jan 21;12(1):112. doi: 10.1038/s41419-020-03350-8 (PMC7819993; doi:10.1038/s41419-020-03350-8)
Supplement: Supplementary file 2 — Additional file 1 Table S1-6 [file 41419_2020_3350_MOESM2_ESM.docx]

**Additional file 1**

**Table S1**

|  | Patient 1 | Patient 2 | Patient 3 |
| --- | --- | --- | --- |
| Age(y) | 63 | 38 | 59 |
| Gender | Female | Male | Female |
| Tumor size(cm) | 4*3.5 | 1*0.8 | 3*2.4 |
| Extrathyroidal extension | Positive | Negtive | Positive |
| Clinical stage | III | I | III |
| T classification | T4a | T1a | T3 |
| N classification | N1b | N1b | N1b |
| Lymph node metastasis  (Positive/Total) | 17+/66 | 15+/52 | 12+/43 |

**Table S2**

| **Primer for qPCR** |  |  |
| --- | --- | --- |
| MiR-296-3p | F | GAGGGTTGGGTGGAGGCTCTCC |
|  | R | Universal R Primer (CWBIO) |
| U6 | F | CTCGCTTCGGCAGCACA |
|  | R | AACGCTTCACGAATTTGCGT |
| RUNX1 mRNA | F | AGCTTCACTCTGACCATCACTG |
|  | R | CTGCCGATGTCTTCGAGGTT |
| CircRUNX1 | F | AGTCAGATGCAGGGGAAAAGC |
|  | R | CTGCCGATGTCTTCGAGGTT |
| β-actin | F | AGAGCTACGAGCTGCCTGAC |
|  | R | AGCACTGTGTTGGCGTACAG |
| GADPH | F | AGGGCTGCTTTTAACTCTGGT |
|  | R | CCCCACTTGATTTTGGAGGGA |
| Human DDHD2 | F | ATGTCATCAGTGCAGTCACAAC |
|  | R | ACTGGTTCATACAAGCTGCCA |
| Human ZNF763 | F | CTCCAGAGCGCATTCTTAGGT |
|  | R | CTCCAGAGCGCATTCTTAGGT |
| Human DEPTOR | F | CTCAGGCTGCACGAAGAAAAG |
|  | R | TTGCGACAAAACAGTTTGGGT |
| Human POSTN | F | GACCGTGTGCTTACACAAATTG |
|  | R | AAGTGACCGTCTCTTCCAAGG |
| Human EGR2 | F | TCAACATTGACATGACTGGAGAG |
|  | R | AGTGAAGGTCTGGTTTCTAGGT |
| Human TUBB4A | F | CCGGACAACTTCGTGTTTGG |
|  | R | TCGCGGATCTTACTGATGAGC |
| Human C1RL | F | CAATCTCATTCGTCGGTTCGG |
|  | R | CAGCCACGGTTTGGTAGAGG |
| Hsa_circ_0061406 | F | CAATAATCGTGCTGCTGACGC |
|  | R | CTTTGTACAGCTGGGTTGCTG |
| Hsa_circ_0008677 | F | AGCGCAAAAAGTTGATCCCC |
|  | R | TTACCATCAGGGGGTGGTCT |
| Hsa_circ_0061407 | F | CACCAGCAACCCAGACACAT |
|  | R | TCGGGCTGTGGAACATAGAG |
| Hsa_circ_0003279 | F | TCTCTGTGCACGACTCTCAGG |
|  | R | CATCTCGTCGTCACTCCACTC |
| Hsa_circ_0076710 | F | TCTTCGCCATGGGATGGGAG |
|  | R | AGAGCCATAGGTCTGGAGGT |
| Hsa_circ_0070659 | F | GCCATCTTCACAGCAGGTTT |
|  | R | GTTACCAAGGGAAGGCCATGT |
| Hsa_circ_0004217 | F | GCAGTCCAAGTCAGCTGTGG |
|  | R | TAGAGCCTGTTCTCGATGGTT |
| Hsa_circ_0061404 | F | CACAGCAGCAAGCTGTACAAAG |
|  | R | GCGTCAGCAGCACGATTATT |
| Hsa_circ_0070074 | F | TTGGGATCTTCACACAGCCAC |
|  | R | CATCTTCCTCAACAGTCTTGGC |

**Table S3**

| **shRNA or siRNAs** |  |
| --- | --- |
| CircSERPINE2 shRNA #1 | GAGUCAGAUGCAGGGGAAA |
| CircSERPINE2 shRNA #2 | AGAGUCAGAUGCAGGGGAATT |
| CircSERPINE2 shRNA #3 | AUGCAGGGGAAAAGCUUCATT |
| DDHD2 si | GATCCATCTCCGTCACCAA |
| DEPTOR si | CCATGTGTGTGATGAGCAT |
| POSTN si | UAAAAAGGGAAUCAUCUUGAG |
| EGR2 si | CGCTAGCCGTCAAGTTCAA |
| ZNF763 si | GGAGAAACTTCAGGAGTCT |
| TUBB4A si | AUAAAUUAGGGCUCAAAGGGG |
| C1RL si | AGUUAUGGGACUCAUUCUGAC |

**Table S4**

| mimics and inhibitors |  |
| --- | --- |
| miR-296-3p mimics | GAGGGUUGGGUGGAGGCUCUCC |
| nc mimics | UUCUCCGAACGUGUCACGUTT |
| miR-296-3p inhibitor | GGAGAGCCUCCACCCAACCCUC |
| Nc inhibitor | CAGUACUUUUGUGUAGUACAA |
| miR-762 mimics | TCTCACTATAAGACGGCTCG |
| miR-1268a mimics | CGGGCGUGGUGGUGGGGG |
| miR-1268b mimics | CGGGCGUGGUGGUGGGUG |
| miR-3147 mimics | GGUUGGGCAGUGAGGAGGGUGUGA |
| miR-4739 mimics | AAGGGAGGAGGAGCGGAGGGGCCCU |
| miR-6846-5p mimics | UGGGGGCUGGAUGGGGUAGAGU |
| miR-6848-5p mimics | UGGGGGCUGGGAUGGGCCAUGGU |

**Table S5**

| **Probes for FISH** |  |
| --- | --- |
| Cy3-circRUNX1 | CY3-GAAGCTTTTCCCCTGCATCTGACTCTG |
| Fam-miR-296-3p | FAM-GGAGAGCCTCCACCCAACCCTC |

| **Table S6** Relationship between circRUNX1 expression and the clinical pathological characteristics of 52 PTC patients | | | | |
| --- | --- | --- | --- | --- |
| Characteristics |  | CircRUNX1 expression | | p Value^†^ |
|  | | Low | High |  |
| Gender | |  |  |  |
| Male | | 8 | 7 | 0.760 |
| Female | | 18 | 19 |  |
| Age (years) | |  |  |  |
| ＜45 | | 14 | 13 | 0.781 |
| ≥45 | | 12 | 13 |  |
| Tumor size (cm) | |  |  |  |
| ＜1 | | 19 | 9 | 0.005^*^ |
| ≥1 | | 7 | 17 |  |
| TNM Stage | |  |  |  |
| I/II | | 22 | 12 | 0.013^*^ |
| III/IV | | 4 | 13 |  |
| Lymph node metastasis | |  |  |  |
| Negtive | | 16 | 8 | 0.026^*^ |
| Positive | | 10 | 18 |  |
| Extra-thyroidal Extension | |  |  |  |
| Negtive | | 23 | 17 | 0.048^*^ |
| Positive | | 3 | 9 |  |
| Nodular Goiter | |  |  |  |
| Negtive | | 18 | 12 | 0.092 |
| Positive | | 8 | 14 |  |
| ^†^Peason’schi-squared test | | | | |
| ^*^p<0.05 | |  |  |  |
