# Supplementary figures and images for: Circular RNA circRUNX1 promotes papillary thyroid cancer progression and metastasis by sponging MiR-296-3p and regulating DDHD2 expression
Source: Cell Death Dis. 2021 Jan 21;12(1):112. doi: 10.1038/s41419-020-03350-8 (PMC7819993; doi:10.1038/s41419-020-03350-8)

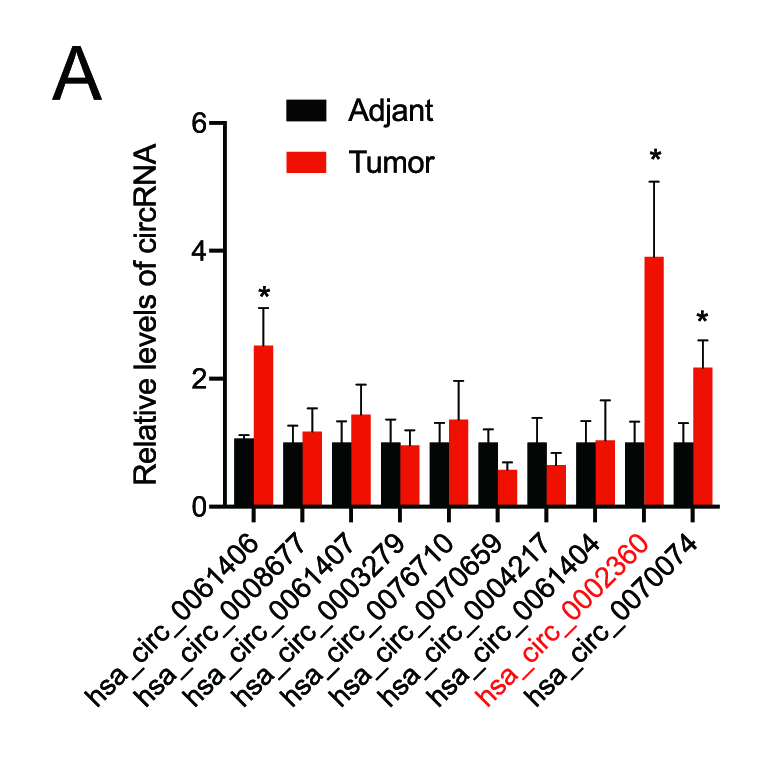

Supplement: Supplementary file 3 — Additional file 2:Figure S1 [file 41419_2020_3350_MOESM3_ESM.tif]

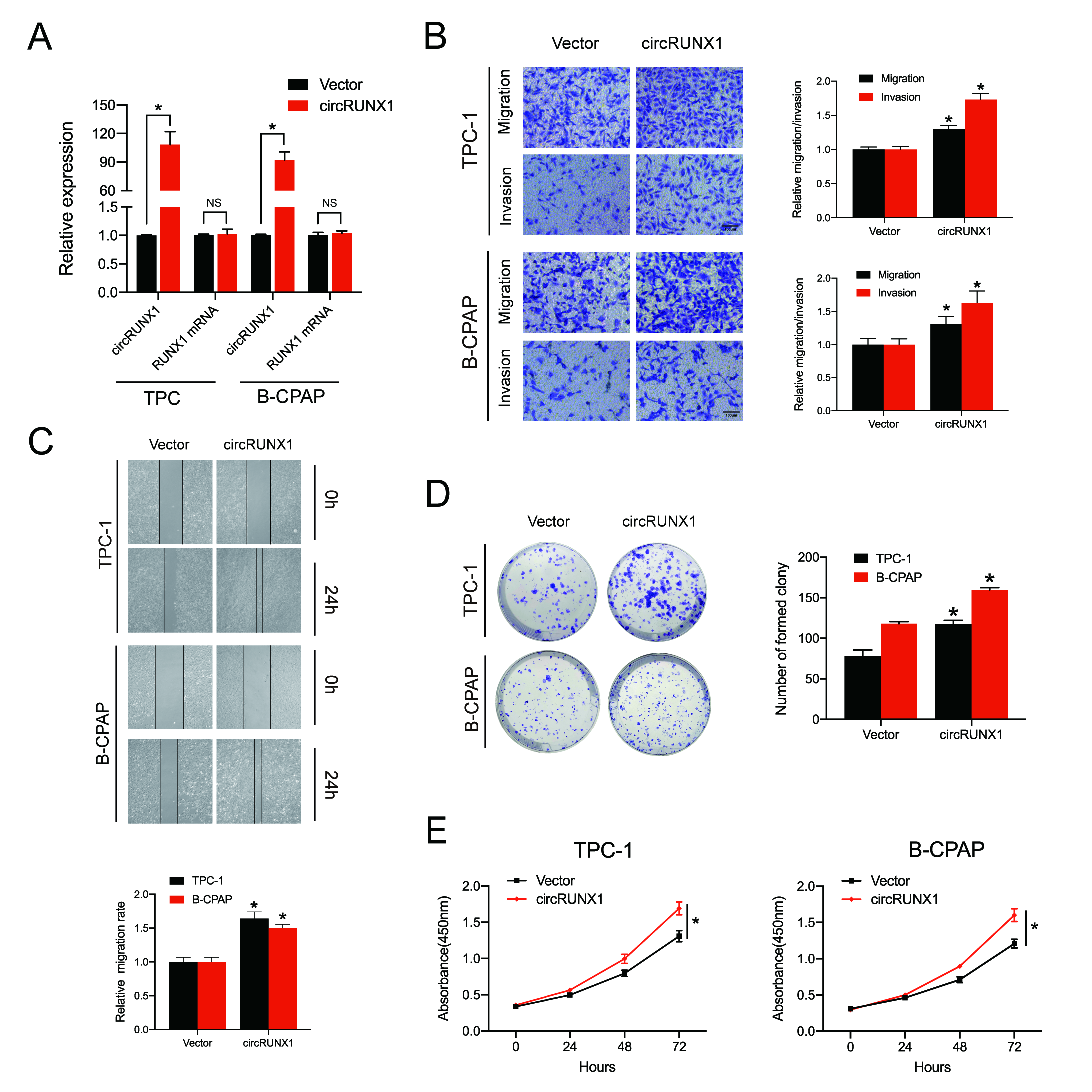

Supplement: Supplementary file 4 — Additional file 3:Figure S2 [file 41419_2020_3350_MOESM4_ESM.tif]

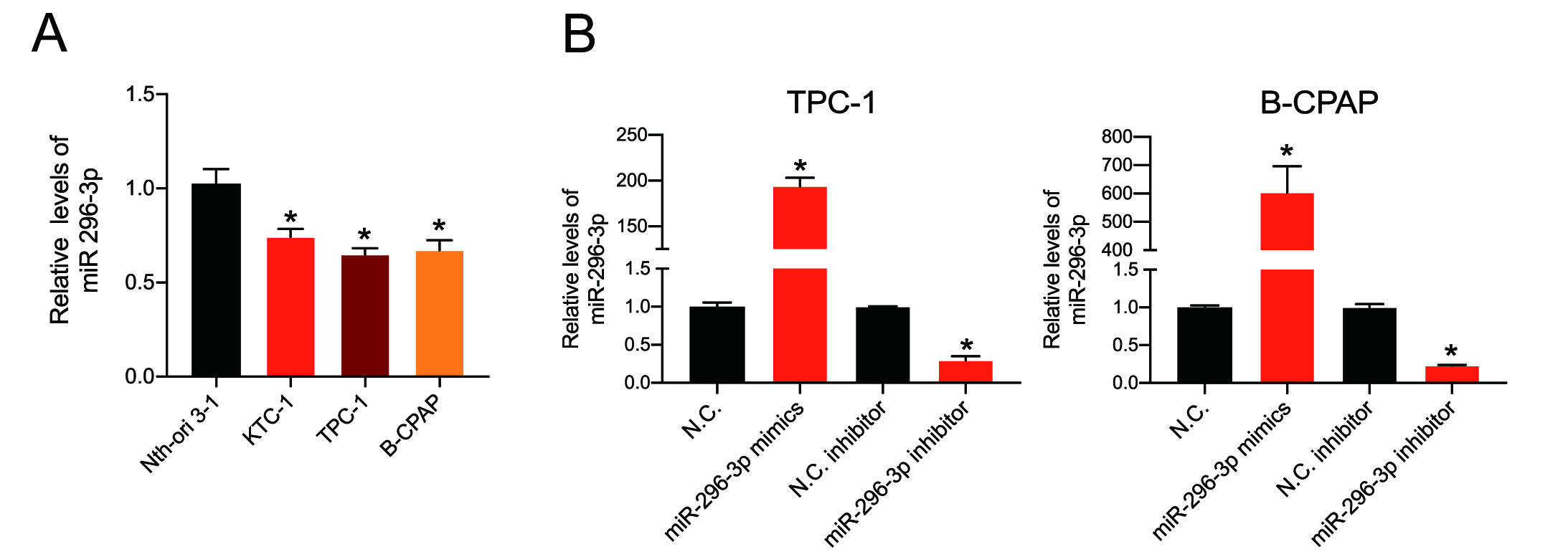

Supplement: Supplementary file 5 — Additional file 4:Figure S3 [file 41419_2020_3350_MOESM5_ESM.tif]

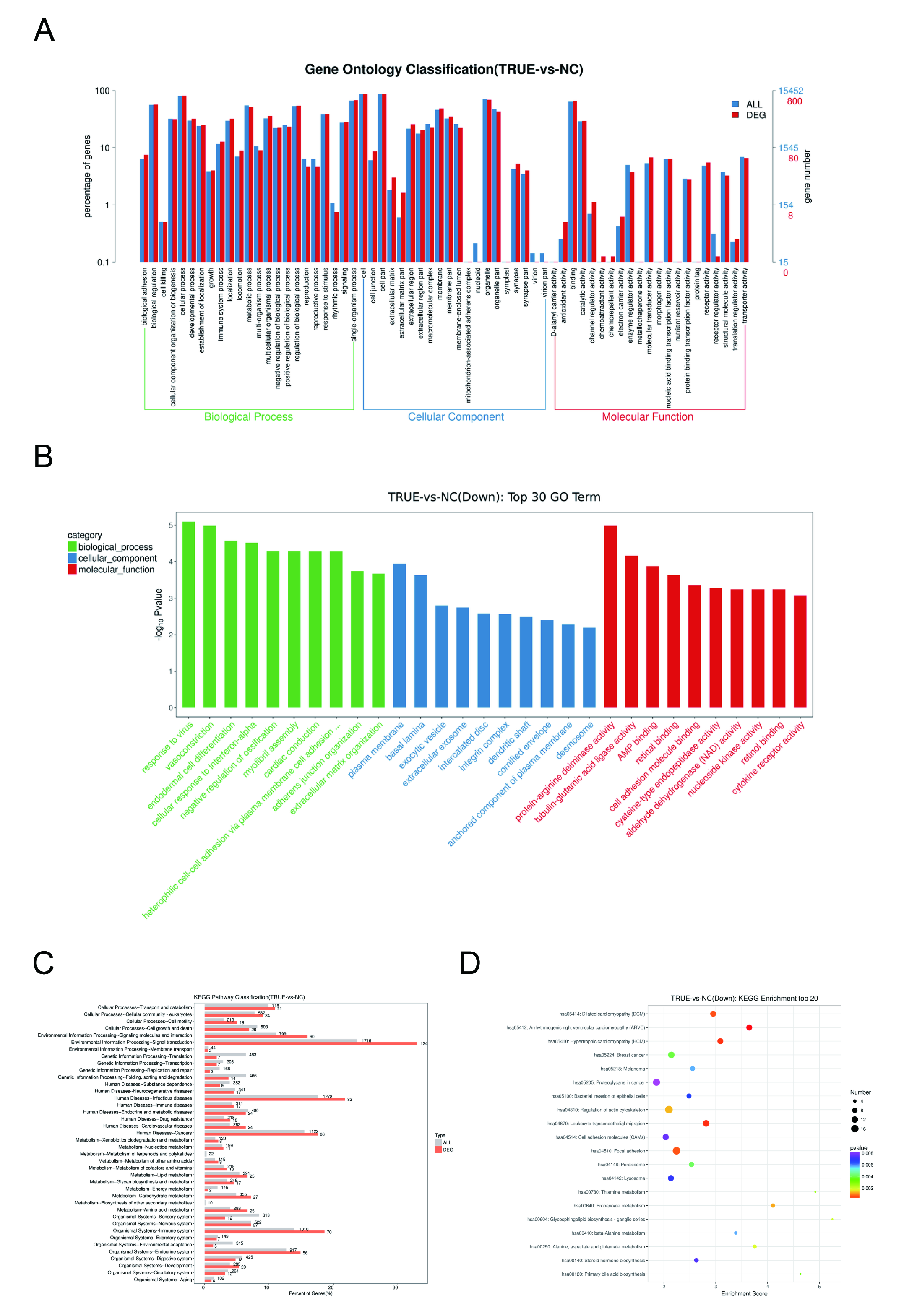

Supplement: Supplementary file 6 — Additional file 5:Figure S4 [file 41419_2020_3350_MOESM6_ESM.tif]

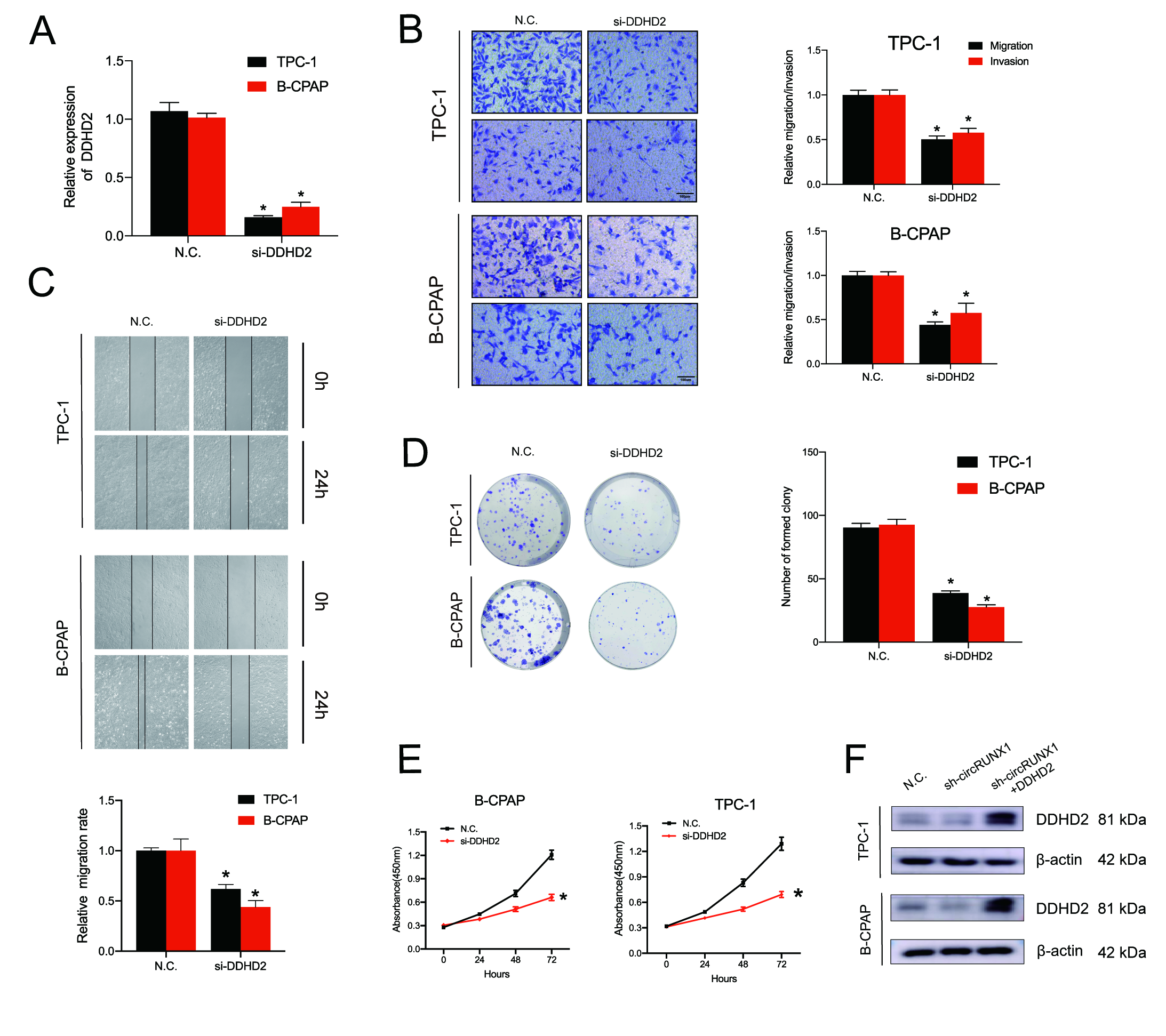

Supplement: Supplementary file 7 — Additional file 6:Figure S5 [file 41419_2020_3350_MOESM7_ESM.tif]
